# Generation and Characterization of Monoclonal Antibodies to the Ogawa Lipopolysaccharide of *Vibrio cholerae* O1 from Phage-Displayed Human Synthetic Fab Library

**DOI:** 10.4014/jmb.2005.05046

**Published:** 2020-08-24

**Authors:** Dain Kim, Jisu Hong, Yoonjoo Choi, Jemin Han, Sangkyu Kim, Gyunghee Jo, Jun-Yeol Yoon, Heesu Chae, Hyeseon Yoon, Chankyu Lee, Hyo Jeong Hong

**Affiliations:** 1 Department of Systems Immunology, College of Biomedical Science, Kangwon National University, Chuncheon 2434, Republic of Korea; 2 Medical Research Center, Chonnam National University Medical School, Hwasun 5818, Republic of Korea; 3 Eubiologics Co., Ltd., Chuncheon 2422, Republic of Korea; 4 Scripps Korea Antibody Institute, Chuncheon 231, Republic of Korea

**Keywords:** *Vibrio cholerae*, Ogawa serotype, O-antigen polysaccharide, monoclonal antibody, phage display

## Abstract

*Vibrio cholerae*, cause of the life-threatening diarrheal disease cholera, can be divided into different serogroups based on the structure of its lipopolysaccharide (LPS), which consists of lipid-A, corepolysaccharide and O-antigen polysaccharide (O-PS). The O1 serogroup, the predominant cause of cholera, includes two major serotypes, Inaba and Ogawa. These serotypes are differentiated by the presence of a single 2-*O*-methyl group in the upstream terminal perosamine of the Ogawa O-PS, which is absent in the Inaba O-PS. To ensure the consistent quality and efficacy of the current cholera vaccines, accurate measurement and characterization of each of these two serotypes is highly important. In this study, we efficiently screened a phage-displayed human synthetic Fab library by bio-panning against Ogawa LPS and finally selected three unique mAbs (D9, E11, and F7) that specifically react with Ogawa LPS. The mAbs bound to *Vibrio cholerae* vaccine in a dose-dependent fashion. Sequence and structure analyses of antibody paratopes suggest that IgG D9 might have the same fine specificity as that of the murine mAbs, which were shown to bind to the upstream terminal perosamine of Ogawa O-PS, whereas IgGs F7 and E11 showed some different characteristics in the paratopes. To our knowledge, this study is the first to demonstrate the generation of Ogawa-specific mAbs using phage display technology. The mAbs will be useful for identification and quantification of Ogawa LPS in multivalent *V. cholerae* vaccines.

## Introduction

*Vibrio cholerae* is a gram-negative bacterium that causes the life-threatening diarrheal disease cholera. *V. cholerae* can be classified into different serogroups based on its lipopolysaccharide (LPS) structure. The LPS has three constituents, lipid-A, core- polysaccharide (core-PS) and O-antigen polysaccharide (O-PS). Lipid A is anchored to the outer membrane, and the core-PS and O-PS are projected outward. Approximately 200 serotypes of *V. cholerae* have been identified based on the O-PS, while serogroups O1 and O139 have been associated with cholera epidemics [1]. Currently, however, the serogroup O139 only accounts for a minority of cholera cases, while serogroup O1 is the predominant cause of cholera [2]. The serogroup O1 includes two major serotypes, Inaba and Ogawa [3]. The O-PS is composed of 12-18 repeating units of α(1-2)-linked 4-amino-4,6-dideoxy-D-mannose (perosamine), the amino group of which is acylated with 3-deoxy-L-glycero-tetronic acid [4, 5]. The upstream perosamine of the O-PS differentiates the Inaba and Ogawa, which differ only by the presence of a single 2-*O*-methyl group in the upstream terminal perosamine unit of the Ogawa O-PS, which is absent in the Inaba O-PS [6-8].

Most *V. cholerae* multivalent vaccines developed so far include the LPSs of Ogawa and Inaba serotypes because these are the main causes of epidemic cholera [9]. Thus, to ensure the consistent quality and efficacy of the current cholera vaccines, methods of accurately identifying and measuring these two serotypes in mixtures is highly important. Enzyme-linked immunosorbent assay (ELISA), which recruits antigen-antibody reactions, is generally used to measure and identify the contents of antigens in multivalent vaccines. To date, monoclonal antibodies (mAbs) directed against *V. cholerae* O1 have been obtained by immunization of mice with the LPS or strain(s) of Ogawa and Inaba serotypes using hybridoma technology [8,10-21]. Most of the mAbs reacted with both Inaba and Ogawa serotypes, due to the minor structural differences in these serotypes, and few of them were Ogawa- or Inaba-serotype specific [8, 14].

The advent of phage display technology has enabled facile isolation of mAbs from combinatorial antibody libraries [22, 23]. The major advantages of phage display include the virtual independence of the selection process from target characteristics, which allows application to a wide range of antigens, irrespective of their immunogenicity or toxicity, and the full control over the selection conditions [24]. The isolation of mAbs by phage display employs a naïve antibody library generated from donor-derived B cells, immune library, a synthetic library from synthetically derived diversity, or a semi-synthetic library in single-chain Fv (scFv) or Fab format [25-31]. ScFv fragments often have a high tendency to form multimers as well as aggregates and have been found to lose affinity during conversion to Ig (immunoglobulin)G, whereas Fab fragments have been found to possess comparably higher structural stability by the presence of the CH1 and light-chain constant domain (CL), and thus their binding activities were retained after conversion to IgG [32]. We previously generated a human synthetic Fab library based on the VH3-23 and VK1-39 framework pairing and have successfully generated mAbs against recombinant proteins and peptide [33-35]

In this study, to generate mAbs against the serotype-specific LPS of *V. cholerae*, we exploited the advantages of phage display technology. We efficiently generated anti-Ogawa mAbs from a human synthetic Fab library and confirmed that they bind to *V. cholerae* vaccine in a dose-dependent fashion. In addition, their fine epitopes and paratopes were characterized through amino acid sequence and structure analyses. The mAbs will be useful for identification and quantification of Ogawa LPS in multivalent *V. cholerae* vaccines.

## Materials and Methods

### Preparation of the LPSs of *V. cholerae*

Isolation and purification of the LPSs from three strains (Ogawa, Inaba, and O139) of *V. cholerae* were performed by the hot phenol-water method [36]. Briefly, the cultivated *V. cholerae* was inactivated by formalin, and then centrifuged at 7,000 ×*g* for 30 min. The cells were washed 3 times with phosphate-buffered saline (PBS) and treated with 45% phenol for 1 h at 68°C. After cooling to 10°C, the water phase (top layer) was obtained by centrifugation at 7,500 ×*g* for 1 h at the same temperature. Ethanol was added to final concentration of 25% (v/v) to remove impurities. LPS was obtained by adding ethanol to make the final concentration of 75% (v/v) and lyophilized to measure weight. Also, the contents of impurities such as protein, nucleic acid and endotoxin were confirmed. The purified Ogawa or Inaba LPS was used as an antigen in panning of the phage-displayed Fab library and in the direct ELISA, while the O139 LPS was used in the indirect ELISA. The antigen specificity of the purified LPS was confirmed by indirect ELISA using the rabbit antiserum (A-FRONTIER, South Korea) to the LPS from the Ogawa, Inaba or O139 strains.

### Bio-Panning of Phage-Displayed Human Synthetic Fab Library

Bio-panning was performed using the phage-displayed human synthetic Fab library (2 × 10^9^ diversity) constructed in our laboratory. Immuno-tubes (Nunc, USA) were coated with the purified Ogawa or Inaba LPS (100 μg/ml) or BSA (10 μg/ml) in coating buffer (Na_2_CO_3_, NaHCO_3_, pH 9.6) overnight at 4°C, washed twice with 0.1% PBST (0.1% Tween 20 in PBS) and blocked by blocking solution (2% skim milk in 0.1% PBST) at 37°C for 1 h. After washing, the phage-displayed human synthetic Fab library was incubated with the BSA at 37°C for 1 h, then unbound phages were recovered and incubated with the Ogawa or Inaba LPS at 37°C for 2 h. After washing five times with 0.1% PBST and twice with PBS, the bound phages were eluted using trypsin (10 μg/ml) in PBS at 37°C for 30 min. Eluted phages were used to infect *E. coli* TG1 cells at optical density (OD) 0.5 at 600 nm, followed by their super-infection with KM13 helper phages [37]. The amplified phages were used for the next round of panning. In the second and third panning, the precleared phages were incubated with the antigen at 10 μg/ml and 1 μg/ml concentrations, respectively, then washed ten times. The input and output phage titers were measured for each round, as described previously [33].

### Screening of Phage-Displayed Fabs (Phage-Fabs) by ELISAs

After three rounds of panning, the TG1 cells from each colony were inoculated at OD 0.1 at 600 nm and cultured in 2 × YT/carbenicillin/glucose broth until reaching OD of 0.5 at 600 nm. The cells were infected with KM13 helper phage at 40 MOI without stirring at 37°C for 30 min, and then with stirring for 30 min. The infected cells were centrifuged at 2,900 ×*g* for 10 min, and the cell pellet was resuspended in 2 × YT/carbenicillin/kanamycin broth and cultured at 30°C with stirring for 12 h. After centrifugation, the supernatant containing phage-Fabs was recovered and analyzed by indirect and quantitative ELISAs.

The phage-Fabs were analyzed by ELISAs as described previously [35]. For the indirect ELISA, the phage-Fabs were incubated with Ogawa, Inaba, O139, or BSA (2 μg/ml) at 37°C for 1 h. After washing, the bound phage-Fabs were incubated with anti-M13 antibody-HRP (1:5000 v/v, GE Healthcare). For the quantitative ELISA, the phage-Fabs were incubated with anti-human kappa antibody (100 ng/well, Thermo Fisher Scientific). After washing, the bound phages were incubated with the anti-M13 antibody-HRP (1:5000 v/v, GE Healthcare). Finally, TMB substrate reagent (BD, OptiEIA) was added and incubated for 5 min. The reaction was stopped with 2.5 M H_2_SO_4_. The absorbance (A) was measured at 450 nm in an ELISA reader (Molecular Devices, USA).

### Conversion of Fabs into IgGs

To convert the selected Fabs into IgG format, the cloning and expression of IgGs were performed as described previously [34]. The IgG was purified from the culture supernatant by affinity chromatography on Protein A-agarose beads (Millipore), and the protein concentration was determined using a NANO-DROP 2000 (Thermo Fisher Scientific). The integrity and purity of the purified antibody were assessed by sodium dodecyl sulfate–polyacrylamide gel electrophoresis (SDS-PAGE).

### Analysis of Antigen-Binding Activities of Purified IgGs by ELISAs

To determine the accurate concentration of each purified IgG, antibody was serially diluted and subjected to quantitative ELISA using anti-human kappa antibody (100 ng/well) and anti-human IgG Fc-HRP (1:8000 v/v, GE Healthcare). To compare the antigen-binding activities of the purified mAbs to different serotypes, various concentrations of each antibody were subjected to indirect ELISA using purified Ogawa, Inaba, or O139 LPS (10 μg/ml) as a coating antigen and anti-human IgG Fc-HRP (1:8000 v/v, GE Healthcare). To evaluate whether the mAbs can be used for quantitation of Ogawa LPS in *V. cholerae* vaccine, each mAb (200 ng/ml) was incubated with serially diluted Euvichol as a coating antigen and an indirect ELISA was conducted. The 96-well microplates (Nunc) were coated with each antigen in coating buffer (Na_2_CO_3_ = 15 mM, NaHCO_3_ = 35 mM, pH 9.6) at 4°C overnight, while all incubations were performed at 37°C for 1 h, as described previously [35]. Finally, the TMB reagent was added and incubated for 6 min.

To analyze the antigen-binding properties of the mAbs for various concentrations of Ogawa LPS, an indirect ELISA was performed as described previously [38]. Briefly, a microtiter plate was coated with Ogawa LPS (0.02 –5 μg/ml) in 10 mM MgCl_2_ in PBS at room temperature overnight. The plate was blocked with 2% skim milk in 0.05% PBST at room temperature for 1 h and washed twice. The coated antigen was incubated with each antibody (200 ng/ml) in 0.1% PBA at room temperature overnight. After washing ten times, the bound antibody was incubated with anti-human IgG Fc-HRP (1:8000 v/v, Thermo Fisher Scientific) at room temperature for 4 h. After washing four times, the TMB reagent was added and incubated for 6 min. Finally, the reaction was stopped to measure the absorbance.

## Results and Discussion

### Bio-Panning of Phage-Displayed Human Synthetic Fab Library

**Fig. 1:**
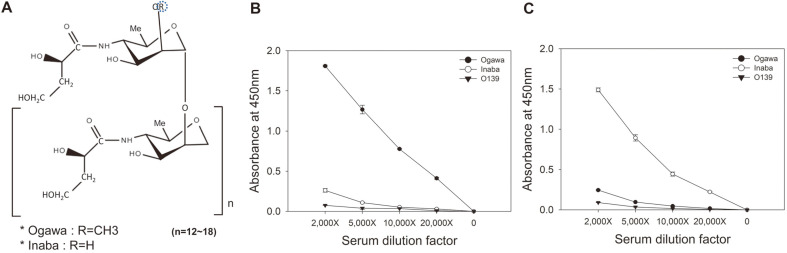
Analysis of antigen specificities of the purified LPSs of Ogawa and Inaba serotypes. (**A**) Chemical structure of the O-PS of *Vibrio cholerae* O1. The O-PS structures of the two serotypes, Inaba and Ogawa, are the same except that at the position O-2 of the upstream terminal perosamine group, R = CH3 in the Ogawa strain and R = H in the Inaba strain, extracted from [43]. (**B** and **C**) Indirect ELISA of the purified Ogawa LPS (**B**) or Inaba LPS (**C**) using serially diluted rabbit antiserum to the LPS of Ogawa (●), Inaba (○), or O139 (▼).

**Fig. 2:**
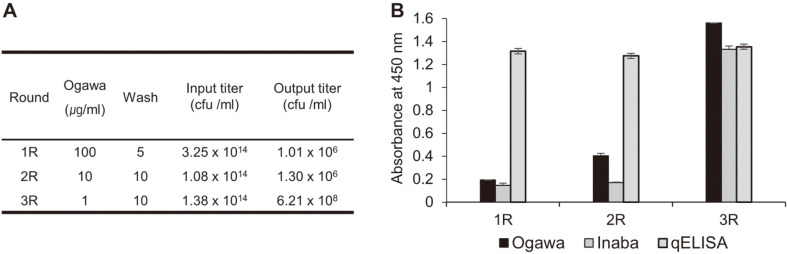
Bio-panning of a phage-displayed human synthetic Fab library against Ogawa LPS. (**A**) Input and output phage titers of each panning round (R). Cfu, colony-forming unit. (**B**) Indirect ELISA and quantitative ELISA of output phage- Fabs from panning against Ogawa LPS. Values were obtained from duplicate wells and are expressed as the mean ± SEM.

We isolated and purified the LPSs of the Ogawa and Inaba serotypes for bio-panning. The structural difference between Ogawa O-PS and Inaba O-PS is shown in Fig. 1A. The antigen specificity of the purified Ogawa or Inaba LPS was confirmed by indirect ELISA using the rabbit antiserum to the LPS of each serotype (Figs. 1B and 1C). Subsequently, three rounds of bio-panning of the phage-displayed Fab library against the purified Ogawa LPS was performed under less stringent washing conditions (5, 10, and 10 times of washing, respectively), compared to that (10, 20, and 30 times of washing, respectively) against protein or peptide antigen, because antibody-carbohydrate interactions are relatively weak compared to antibody-protein (or peptide) interaction [8, 39, 40]. The output phage titers largely increased after the third round of panning (Fig. 2A), but the enriched polyclonal phage-Fabs contained the clones that reacted with both Ogawa and Inaba LPSs (Fig. 2B).

### Selection of Anti-Ogawa Fabs

**Fig. 3:**
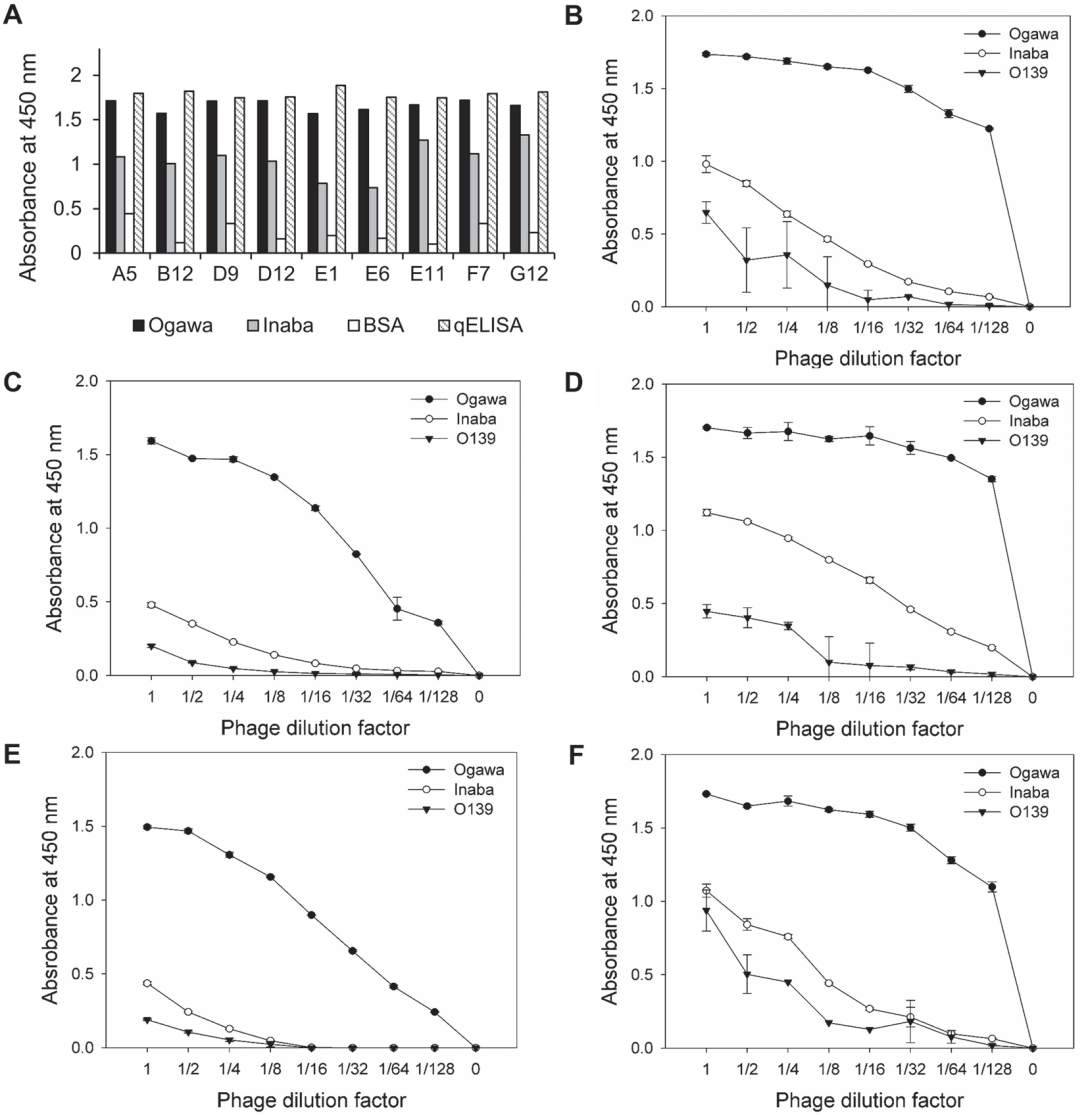
Analysis of antigen-binding activities of anti-Ogawa phage-Fabs. (**A**) Indirect ELISA and quantitative ELISA of nine unique phage-Fabs. The phage-Fabs were incubated with Ogawa LPS, Inaba LPS, or BSA (200 ng/well) at 37°C for 1 h for indirect ELISA or anti-human kappa antibody (100 ng/well) for quantitative ELISA (qELISA). The bound phages were incubated with anti-M13 antibody-HRP. (B–F) D9 (**B**), D12 (**C**), F7 (**D**), E11 (**E**) or A5 (**F**) phage-Fabs were serially diluted and subjected to indirect ELISA using Ogawa LPS (●), Inaba LPS (○), or O139 LPS (▼) as a coating antigen.

**Fig. 4:**
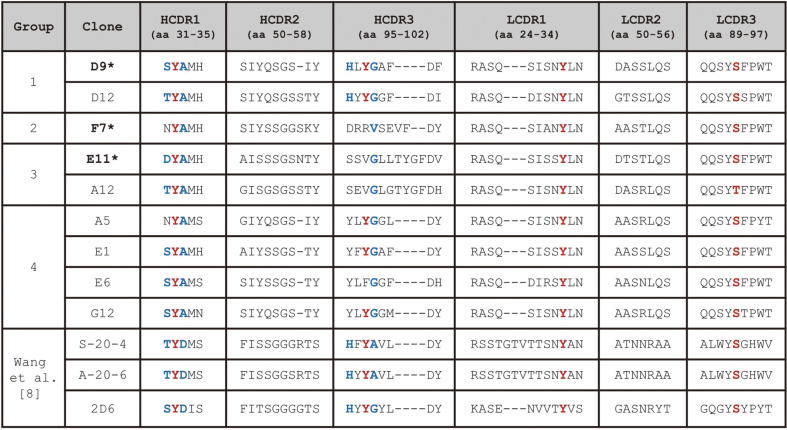
Comparison of the amino acid sequences of the three heavy- or light-chain CDRs between the nine unique anti-Ogawa mAbs and the previous three murine mAbs. The nine unique anti-Ogawa Fabs were classified into four different groups, based on the lengths and sequence homology of HCDR3. The CDR sequences of the three murine mAbs (S-20-4, A-20-6, and 2D6) were excerpted from Wang *et al*. [8]. Asterisk mark (*) denotes the Fab clone whose antigenbinding activity was determined by ELISAs, while the IgGs whose antigen-binding activities were determined are shown in bold. Amino acids (aa) are numbered according to Kabat. A dash indicates no amino acid residue in the position. The four epitope-binding residues in the HCDR1 and HCDR3 are colored in blue, and the four “fence” amino acids that were shown to form a distinct hydrophobic binding pocket are colored in red. The S-20-4 and A-20-6 have λ light chains, whereas the other mAbs have κ light chains.

To select the Fabs specific for Ogawa LPS, 96 colonies were randomly selected, and phage-Fabs were screened by indirect ELISA using the Ogawa or Inaba LPS and quantitative ELISA using anti-kappa antibody. This allowed ranking of the positive clones in order of antigen-binding activity. Finally, eleven clones with the highest Ogawa-binding activities and lowest Inaba-binding activities were selected, then nine unique clones were obtained after DNA sequencing (Figs. 3A and Fig. 4).

### Sequence Analysis and Antigen-Binding Specificities of Unique Anti-Ogawa Fabs

The heavy-chain variable region (VH) or light-chain variable region (VL) of an antibody molecule contains three complementarity-determining regions (CDR1, CDR2 and CDR3) that mediate specific binding to different antigens. Among these, heavy-chain CDR3 (HCDR3) and light-chain CDR3 (LCDR3), formed by V-(D)-J joining and V-J joining, respectively, largely contribute to antibody diversity [41]. Sequence analysis of the nine unique anti-Ogawa Fabs revealed that they could be classified into four different groups, based on the lengths and sequence homology of HCDR3 (Fig. 4). For characterization of antigen-binding specificities, the phage-Fabs of five unique clones (D9, D12, F7, E11, and A5), representing the four different groups, were selected and their antigen-binding activities to the LPS of Ogawa, Inaba, or O139 were measured by indirect ELISA (Figs. 3B–3F). All of the Fabs bound to the Ogawa LPS with high activities but produced very low-level reactions with the Inaba LPS, while the phage titer of D9, F7 or A5 was higher than that of D12 or E11. Finally, three unique Fab clones (D9, F7, and E11) were selected for further characterization.

### Conversion of Anti-Ogawa Fabs into IgGs and Analysis of Antigen-Binding Activities

**Fig. 5:**
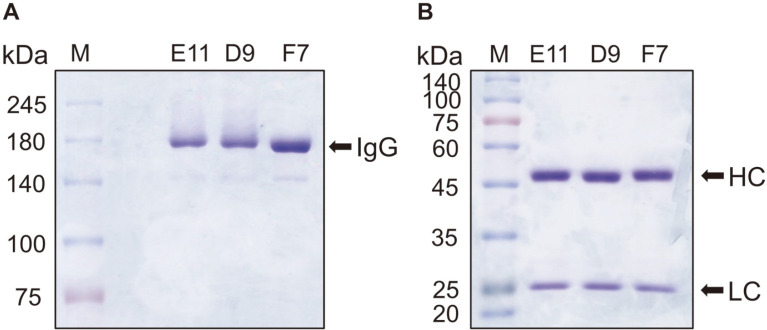
SDS-PAGE analysis of three purified anti-Ogawa IgGs. (**A**) 6% SDS-PAGE under non-reducing condition. (**B**) 10% SDS-PAGE under reducing condition.

The three unique Fabs were converted into IgGs and these were expressed transiently in HEK293F cells. The secreted IgG in culture supernatants was purified by affinity chromatography using Protein A agarose beads, and the purity and integrity of the purified antibody was confirmed by SDS-PAGE under the unreduced and reduced conditions (Fig. 5).

**Fig. 6:**
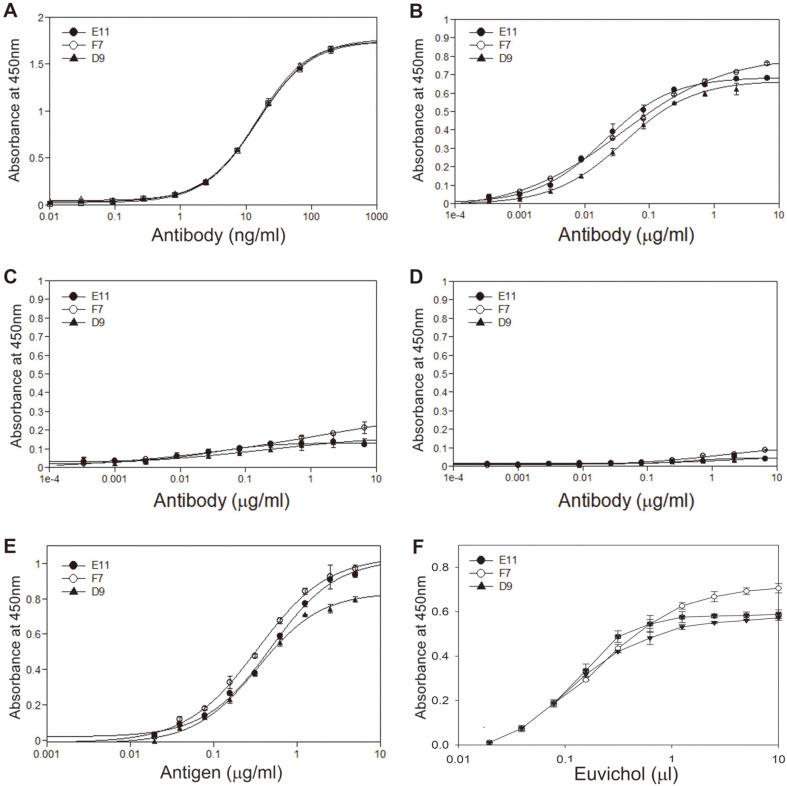
Analysis of antigen-binding activities of purified anti-Ogawa IgGs by ELISAs. (**A**) Quantitative ELISA of purified IgG (D9, E11, or F7) at the concentrations (0.01–200 ng/ml) using anti-human kappa antibody and anti-human IgG Fc-HRP. (**B**–**D**) Indirect ELISA of 3-fold increasing concentrations (0.0005–6.561 μg/ml) of IgG using the Ogawa LPS (**B**), Inaba LPS (**C**), or O139 LPS (**D**) at 10 μg/ml concentration as a coating antigen. One μg/ml of antibody concentration corresponds to 6.667 nM. (**E**) Indirect ELISA of IgG (200 ng/ml) using various concentrations (0.02–5 μg/ml) of Ogawa LPS as a coating antigen. (**F**) Indirect ELISA of IgG (200 ng/ml) using serially diluted *V. cholerae* vaccine (0.02–10 μl) as a coating antigen. Values were obtained from duplicate wells and are expressed as the mean ± SEM.

To verify antigen specificity of the three IgGs (D9, E11, and F7), the accurate concentration of each purified IgG was determined by quantitative ELISA (Fig. 6A) and their antigen-binding activities to the purified Ogawa, Inaba, or O139 LPS (10 μg/ml) were measured by indirect ELISA. The three IgGs bound to Ogawa LPS in a dose-dependent fashion, while IgGs E11 and F7 exhibited slightly higher antigen-binding activities in comparison to IgG D9 (Fig. 6B). In contrast, they hardly reacted with Inaba or O139 LPS, although F7 at the highest concentration exhibited low reactivity to Inaba LPS (Figs. 6C and 6D).

Next, to analyze the antigen-binding properties of the mAbs for various concentrations of Ogawa LPS, each mAb (200 ng/ml) was incubated with serially diluted antigen and an indirect ELISA was performed. As shown in Fig. 6E, the mAbs reacted with Ogawa LPS at 0.02–1.25 μg/ml concentration in a dose-dependent fashion, while F7 showed slightly higher antigen-binding activity compared to D9 or E11. Finally, to evaluate whether the mAbs can be used for quantitation of Ogawa LPS in *V. cholerae* vaccine, each mAb (200 ng/ml) was incubated with serially diluted Euvichol as a coating antigen and the indirect ELISA was performed (Fig. 6F). The mAbs showed good reactivity to the vaccine in a dose-dependent manner, while F7 at high concentrations (2.5–10 μg/ml) exhibited higher antigen-binding activity compared to D9 or E11. This result suggests that the mAbs can be used for identification and quantification of Ogawa LPS in multivalent *V. cholerae* vaccines.

### Sequence and Structure Analyses of Ogawa-Specific mAbs for Characterization of Fine Epitopes

Wang *et al*. reported that three Ogawa-specific murine mAbs, S-20-4 (IgG, λ), A-20-6 (IgG, λ), and 2D6 (IgA, κ), obtained by immunization of mice with the LPS of Ogawa, exhibited the same fine specificity [8]. Thereafter, crystallographic study of Fab S-20-4 in complex with synthetic analogs of Ogawa O-PS showed that the four HCDR residues (Thr31 and Asp33 in the HCDR1 and His95 and Ala98 in the HCDR3) in the antibody are in direct contact with upstream terminal perosamine of the Ogawa O-PS (Fig. 1A), whereas few direct contacts were observed between the antibody paratope and the Ogawa-specific 2-*O*-methyl group [42].

To gain insight into the epitope specificities of our human anti-Ogawa IgGs, the VH and VL sequences of the mAbs were compared with those of the three murine mAbs (Fig. 4). Interestingly, the four HCDR residues (Thr31, Asp33, His95, and Ala98) in S-20-4, which interact with the upstream terminal perosamine, are conserved in D9 or D12, except that Asp33 in the former was replaced by Ala33 in the latter (Fig. 4). Especially, the three HCDR residues (Ser31, His95, and Gly98) in D9 are identical to those in 2D6. Also, the length of HCDR3 in D9 or D12 is the same as that in the three murine mAbs. The sequence analysis suggested that IgG D9 or D12 might bind to the same fine epitope as those of the murine mAbs.

**Fig. 7:**
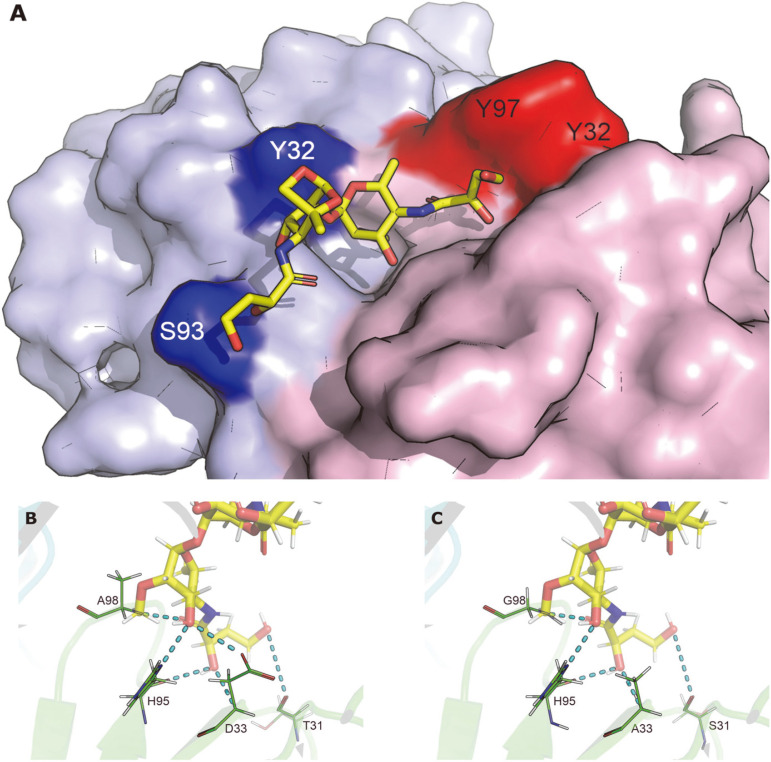
Structural analysis of anti-Ogawa IgGs. (**A**) The O-PS disaccharide epitope binds to the hydrophobic pocket of S-20-4 antibody (PDB ID 1F4Y). There are four residues which form a “fence” of the binding pocket. Two HCDR residues (Tyr32 and Tyr97) and two LCDR residues (Tyr32 and Ser93) are colored in red and blue, respectively. (**B**) The epitope forms six hydrogen bonds with S-20-4 antibody, including two from side chains (Asp33 and His95). (**C**) Five of the six hydrogen bonds remain the same for D9 antibody. Though the substitution D33A may result in the loss of a hydrogen bond, no steric clash may occur and thus the binding should be maintained upon the mutation.

Regarding the structural analysis of the antibody paratope, the structure of S-20-4 and O-PS complex (PDB ID: 1F4Y) shows that the CDRs of the antibody have a distinct hydrophobic binding pocket which structurally fits the ligand (Fig. 7A). The binding pocket is surrounded by four “fence” amino acids (Tyr32 in HCDR1, Tyr97 in HCDR3, Tyr32 in LCDR1 and Ser93 in LCDR3) which are highly conserved in D9 and D12 (and other mAbs of group 4) (Fig. 4), indicating that their binding pockets are also structurally conserved. In addition, a close look into the four HCDR residues (Thr31, Asp33, His95, and Ala98) in S-20-4 shows that they form six hydrogen bonds with the epitope, including two from the side chain interactions in Asp33 and His95 (Fig. 7B). Likewise, in silico mutations and structural analyses of D9 were performed using PyMol. Though three of the four amino acids are different between S-20-4 and D9 (T31S, D33A, and A98G), hydrogen bonds of D9 remain nearly identical, except for the loss of one hydrogen bond due to D33A (Fig. 7C). Taken together, the data from the sequence and structure analyses show that IgG D9 or D12 may bind to the same fine epitope as those of the murine mAbs.

On the other hand, IgGs F7 and E11, which exhibited slightly higher antigen-binding activities compared to D9 in Figs. 6B ando not possess all of the four “fence” amino acids nor the four HCDR residues binding to the epitope. Also, they have longer HCDR3 in comparison with D9 (Fig. 4). It will be interesting to explore how F7 and E11 bind to Ogawa O-PS. Finally, we speculate that the use of phage-displayed synthetic Fab library allowed the isolation of these new mAbs, demonstrating the advantage of phage display technology.

In conclusion, we efficiently generated three groups of new human mAbs that bind to Ogawa LPS from a human synthetic Fab library. One group (IgG D9 or D12) may bind to the same epitope as those of previously reported murine mAbs, and the other two groups of mAbs (IgG F7 and E11) need further characterization regarding their fine epitopes. To our knowledge, this study is the first to show the generation of Ogawa O-PS-specific mAbs from a phage-displayed antibody library. The mAbs will be useful for identification and quantification of Ogawa LPS in multivalent *V. cholerae* vaccines.
